# HIV Progression Depends on Codon and Amino Acid Usage Profile of Envelope Protein and Associated Host-Genetic Influence

**DOI:** 10.3389/fmicb.2017.01083

**Published:** 2017-06-15

**Authors:** Ayan Roy, Rachana Banerjee, Surajit Basak

**Affiliations:** ^1^Department of Botany, Bioinformatics Facility, University of North BengalSiliguri, India; ^2^Structural Biology and Bio-Informatics Division, CSIR-Indian Institute of Chemical BiologyKolkata, India; ^3^Department of Molecular Biology and Bioinformatics, Tripura UniversityAgartala, India; ^4^Bioinformatics Centre, Tripura UniversityAgartala, India

**Keywords:** rapid progressor, slow progressor, long-term non-progressor, evolutionary rate, disease progression

## Abstract

Acquired immune deficiency syndrome (AIDS) is a spectrum of conditions caused by infection with the human immunodeficiency virus (HIV). Two types of HIV have been characterized: HIV-1 and HIV-2. The present study investigated whether evolutionary selection pressure differs between rapid progressor (RP), slow progressor (SP), and long-term non-progressor (LTNP) of HIV-I infected individuals. An unexpected association between the evolutionary rate of substitution in envelope (env) gene and disease progression is observed. Our present study suggests that env genes of LTNP are subject to unusually strong functional constraint with respect to RP. We also observed that the three categories of env genes i.e., RP, SP, and LTNP, had their own characteristic pattern of amino acid usage and SP and LTNP sequences shared similar patterns of amino acid usage different from RP sequences and evolutionary rate significantly influenced the amino acid usage pattern of the three different types of env gene sequences. It was also noted that the evolutionary rate for the glycosylation sites of LTNP and SP sequences were even significantly less than the RP sequences. Comparative analysis on the influence of human host on the three categories of env genes are well correlated with the rates of disease progression suggesting the adaptive strategies of the viruses for successful residence and infection. Host associated selective constraints appeared most relaxed on the RP sequences and strongest in LTNP sequences. The present study clearly portrays how evolutionary selection pressure differs between three categories of env genes i.e., RP, SP, and LTNP. The env genes, coding for the env glycoproteins, experience severe selection constraints from the host due to their constant exposure to the host immune system. In this perspective it might be suggested that env gene evolution occurs mainly by negative selection with the occurrence of mutation that might not reach fixation in the viral population. This work also confers a deeper insight into the crucial effects of host factors that govern the overall progression of HIV infection.

## Introduction

Human immunodeficiency viruses (HIVs) are one of the most crucial members of the retroviral family *Retroviridae*. Among the two main types of HIVs i.e., HIV-1 and HIV-2, HIV-1 has been characterized as the major causative agent of acquired immune deficiency syndrome (AIDS) (Blattner et al., 1988; Weiss, 1993).

Natural variability of HIV-1 is the key underlying the complex biology of the menacing virus. Evolutionary and epidemiological history of HIV can be investigated through phylogenetic analysis of erratic forms of the virus. Interestingly, due to rapid availability of nucleotide sequence data of viral genes, it is nowadays possible to illustrate a detailed phylogenetic relationship of viruses like HIV. Phylogenetic arrangements of HIV-1 are commonly carried out using nucleotide sequences of different sub-genomic regions of the same HIV-1 genome i.e., gag, pol, and envelope (env). This approach has already revealed unique inter-subtype recombinant forms of virus isolates (McCutchan, 2006). Envelope (env) gene encodes known targets for cytotoxic T lymphocytes and neutralizing antibodies (Goudsmit et al., 1988; Chesebro et al., 1991). Despite extensive study of sequence variation of env gene of HIV-1, the exact impact of selection in governing the patterns of variation in the env gene still remains somewhat obscure. In some earlier studies, based on lower frequency of synonymous nucleotide substitutions within the V3 loop in the env genes, it has been circuitously verified that the variation in env gene is maintained by selection for antigenic diversity (Simmonds et al., 1991; Bonhoeffer et al., 1995). Theoretically, it has also been implicated that selection pressure and mutability dynamics are the vital forces that affect viral fitness and robustness (Brown, 1997). It has been hypothesized that negative (purifying) selection constraints, operational in HIV-1, execute a crucial role in devising molecular evolutionary patterns of HIV-1 in contrast to positive (diversifying) selection which has been reported to play a minor role (Seo et al., 2002; Drummond et al., 2003).

Investigations pertaining to viral divergence in HIV-1 patients, associated with varying rates of progression of disease, have often produced interesting and conflicting results (Ganeshan et al., 1997; Arts and Quiñones-Mateu, 2003; Rangel et al., 2003). Some researchers have noted that non-synonymous substitutions in the env gene have been higher in long-term non-progressor (LTNP) whereas, there has been no difference between slow and normal progressors (NPs) regarding the influence of synonymous substitution (Strunnikova et al., 1995; Bagnarelli et al., 1999). However, Markham and co-workers observed opposite results with greater accumulation of non-synonymous substitutions in NPs (Markham et al., 1998). The event of T-cell activation can be effectively employed to estimate the progression to AIDS in HIV-1 infected individuals and might be utility in apt assessment of viral replication rates and evolutionary complexities. Consequently, activation of immune responses might enforce critical constraints on the evolutionary tricks and viral generation rates of HIV. However, such effects yet remain unexplored from the evolutionary perspective. Extensive investigations pertaining to evolutionary signatures might prove handy in elucidating the patterns of molecular adaptation of HIV in human host. Variations in synonymous substitution rates reflect changes in generation time or mutation rate, while rate of non-synonymous mutations tend to be affected by changes in selective pressure and effective population size (Lemey et al., 2007).

Envelope (env) gene is the fastest evolving one in the HIV-1 genome. Conflicting selective pressures shape the evolutionary dynamics of the virus (Korber et al., 2000; Ross and Rodrigo, 2002; Yoshida et al., 2011). In the present study, investigations pertaining to the evolutionary traits of the env genes representing rapid progressor (RP), slow progressor (SP), and LTNP human patients (available in public domain), have been performed extensively through detailed phylogenetic analysis, followed by subsequent estimation of synonymous and non-synonymous substitution rates. Rapid-progressors are associated with speedy development of AIDS within 3 years of infection whereas; slow-progressors are characterized by a comparatively slower gradual onset of AIDS after seroconversion that might take a span 3–10 years (Kumar, 2013) to develop. Comparative analysis of relative synonymous codon usage (RSCU) patterns of the three categories of env genes marked by varying degrees of disease progression i.e., RP, SP, and LTNP types and their similarity index with human host has also been executed in the present endeavor with a motif to address the adaptive strategies of the viruses for successful residence and infection. Results pertaining to RSCU patterns and evolutionary dynamics might prove utility to unravel the molecular underpinnings of viral adaptation and infection in human host and excavate subtle discrepancies among the viral types associated with varying rates of disease progression.

## Materials and methods

### Sequence retrieval

All available env gene sequences of HIV-1 subtype B were retrieved from HIV Database (http://www.hiv.lanl.gov/) representing three types of patient categories i.e., RP, SP, and LTNP patients. Redundant and erroneous sequences (sequences with internal stop codons) were removed to avoid stochastic variations and sampling errors (Wright, 1990). A comprehensive set of 264 coding sequences constituted final dataset for our analysis (Supplementary Material, Data Sheet 1).

### Phylogenetic analysis

Phylogenetic analysis provides the ancestral relationship of a set of sequences. It involves the construction of a tree, where the nodes indicate separate evolutionary paths, and the lengths of the branches give an estimate of how distantly related the sequences represented by those branches are. In the present study, all the env genes were aligned using the Clustal Omega program (https://www.ebi.ac.uk/Tools/msa/clustalo/). The resultant multiple sequence alignments were subsequently used to construct the neighbor-joining method based phylogenetic tree with 500 bootstrap replicates. MEGA 7 was used for phylogenetic analysis (Kumar et al., 2016). Here, we have constructed an unrooted tree, where, the distances and relationships between the taxa have been plotted without making any assumption concerning their descent.

### Estimation of relative synonymous codon usage

Relative synonymous codon usage (RSCU) (Sharp et al., 1986) is calculated as the ratio of the observed frequency of a codon to the expected frequency if codon usage was uniform within a synonymous codon group.

Many genes display a non-random usage of synonymous codons for specific amino acids. A measure of the extent of this non-randomness is given by the RSCU (Sharp and Li, 1986). It is the ratio of observed frequency of the codons with respect to the expected frequency of the same codon if codon usage was uniform within a synonymous codon group.

RSCU is calculated as:

$$RSCU=\frac{Frequency\;of\;codon}{\begin{array}{l} Expected\;frequency\;of\;codon\\ \left(if\;codon\;usage\;was\;uniform\right) \end{array}}$$

Relative synonymous codon usage (RSCU) values > 1 indicate that the observed frequency of synonymous codons is more compared to the expected frequency and lower than one indicates the opposite (dos Reis et al., 2003).

RSCU values of the 59 codons [excluding the single synonymous codons AUG (Met) and UGG (Trp) and the three termination codons] of RP, SP, and LTNP env gene sequences were calculated using CodonW (Ver. 1.4.2) software (http://www.molbiol.ox.ac.uk/cu) (Peden, 2000). Codon usage frequencies of human host (*Homo sapiens*) were obtained from the Codon Usage Database (http://www.kazusa.or.jp/codon/) (Nakamura et al., 2000).

### Assessment of similarity index

Viral genomes are relatively smaller in size and largely rely on the host to execute crucial biological activities like replication, protein synthesis and transmission (Nasrullah et al., 2015). In this pretext, it has been suggested that viral robustness, survival and evasion of host's immune signals and responses largely depend on the interplay of codon usage patterns of the concerned virus and its respective host (Shackelton et al., 2006; Moratorio et al., 2013). Here, we have considered the similarity index to understand the influence of host genome on the adaptability of virus genome inside the host. The influence of the overall codon usage pattern of the host on the formation of the overall codon usage of the virus is defined as the similarity index.

Relative synonymous codon usage (RSCU) values of the three different types of env gene sequences i.e., RP, SP, and LTNP were compared with that of human host in order to assess the influence of human host system in shaping the patterns of codon usage among the env types. The parameter similarity index, *D(A,B)* (Nasrullah et al., 2015) is computed as follows:

$$\begin{array}{l} R(A,B)=\frac{\sum_{i\;=\;1}^{i=59}a_{i}Xb_{i}}{\sqrt{\sum_{i\;=\;1}^{i\;=\;59}a_{i}^{2}X\sum_{i\;=\;1}^{i\;=\;59}b_{i}^{2}}}\\ D(A,B)=\frac{1-R(A,B)}{2} \end{array}$$

where *R(A,B)* refers to the cosine value of an included angle between A and B spatial vectors and represents the similarity between particular env type of HIV and overall codon usage pattern of human host. *a*_*i*_ signifies the RSCU value for a particular codon among the pool of 59 codons for every specific type of env (i.e., RP, SP, and LTNP) protein coding sequence. *b*_*i*_ indicates the RSCU value for the same codon in case of human host. *D(A,B)* signifies the probable impact of human codon usage patterns on the concerned env types of HIV. The value of similarity index, *D(A,B)*, has been reported to lie between 0 to 1.0 (Zhou et al., 2013).

### Multivariate analyses on amino acid usage

Correspondence analysis (COA) (Peden, 2000; http://www.molbiol.ox.ac.uk/cu) was used to investigate the major trend in amino acid usage variation among the env genes. Since amino acid usage by its very nature is multivariate, it is necessary to analyse this data with multivariate statistical techniques i.e., COA. Correspondence analysis (COA) is an ordination technique that identifies the major trends in the variation of the data and distributes genes along continuous axes in accordance with these trends. It has the advantage of not to make any assumption that the data falls into discrete clusters and therefore represent continuous variation accurately. Correspondence analysis (COA) on relative amino acid usage (RAAU) of env gene sequences was executed employing the CodonW program.

### Evolutionary rate calculation

The ratio (ω) of rate of non-synonymous substitutions per non-synonymous site (Ka) to rate of synonymous substitutions per synonymous site (Ks) indicates the impact of evolution on a gene segment. ω > 1 indicates diversifying (positive) selection whereas, ω < 1 signifies purifying (negative) selection (Roy et al., 2015). The evolutionary rates of the orthologous env genes representing all three types under analysis i.e., RP, SP, and LTNP (with reference to progressors) were calculated using Codeml program included in the PAML software package (ver. 4.5) (Nei and Gojobori, 1986; Yang, 2007) (http://abacus.gene.ucl.ac.uk/software/paml.html) with runmode = −2 and CodonFreq = 1.

Evolutionary rate of each individual residue for a given env gene sequence was calculated using SWAKK server (http://ibl.mdanderson.org/swakk/) (Liang et al., 2006). It estimates the ratio of non-synonymous to synonymous substitution rates (Ka/Ks) between a pair of protein-coding DNA sequences, by a sliding 3D window analysis.

## Results

### Phylogenetic profiling of various categories of env genes

**Figure 1:**
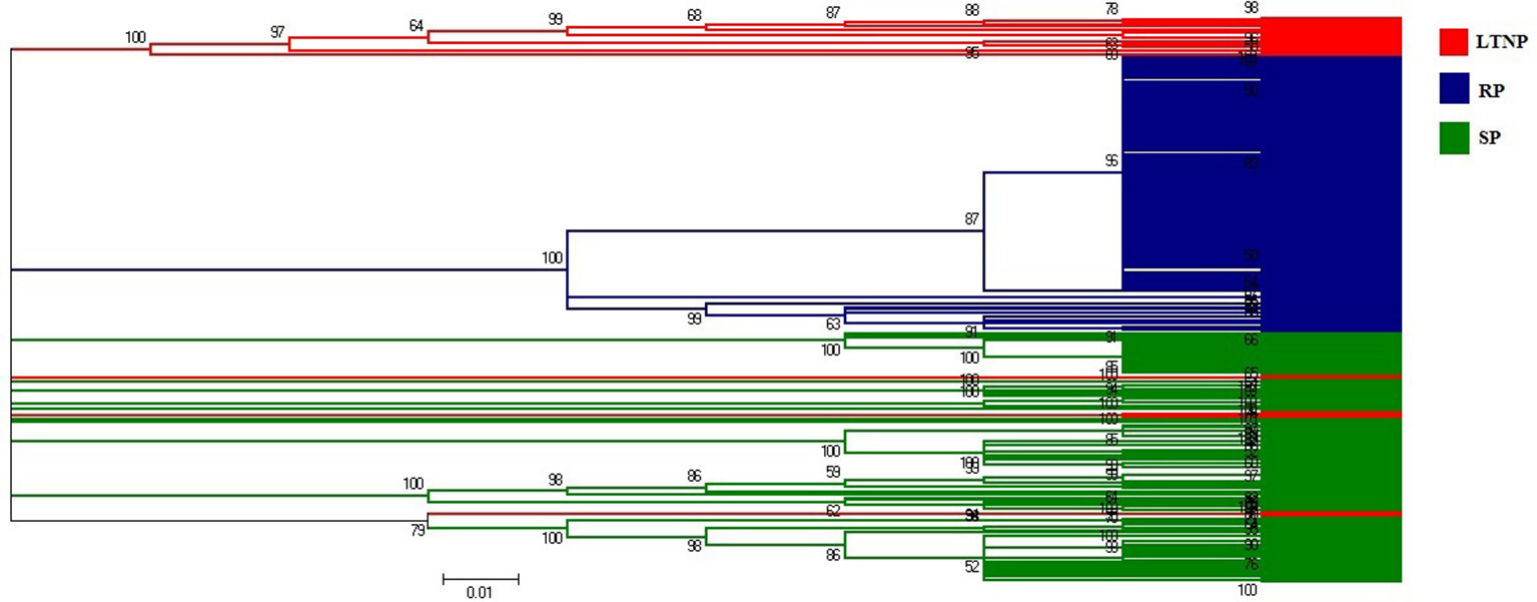
Neighbor-joining method based phylogenetic tree of the env genes (LTNP, SP, and RP). Red colored lines represent Long term non-progressor (LTNP) sequences. Blue colored lines refer to the Rapid progressor (RP) sets. Green colored lines depict the Slow progressor (SP) sequences.

Phylogenetic analysis was performed to investigate the evolutionary relationship of 264 env gene sequences of HIV-1 subtype B, representing three different categories of patients (RP, SP, and LTNP). The phylogenetic tree revealed three major lineages where mostly three categories of env gene sequences i.e., RP, SP, and LTNP have been clustered (Figure 1).

### Trends in amino acid usage of env genes representing varying rates of disease progression

**Figure 2:**
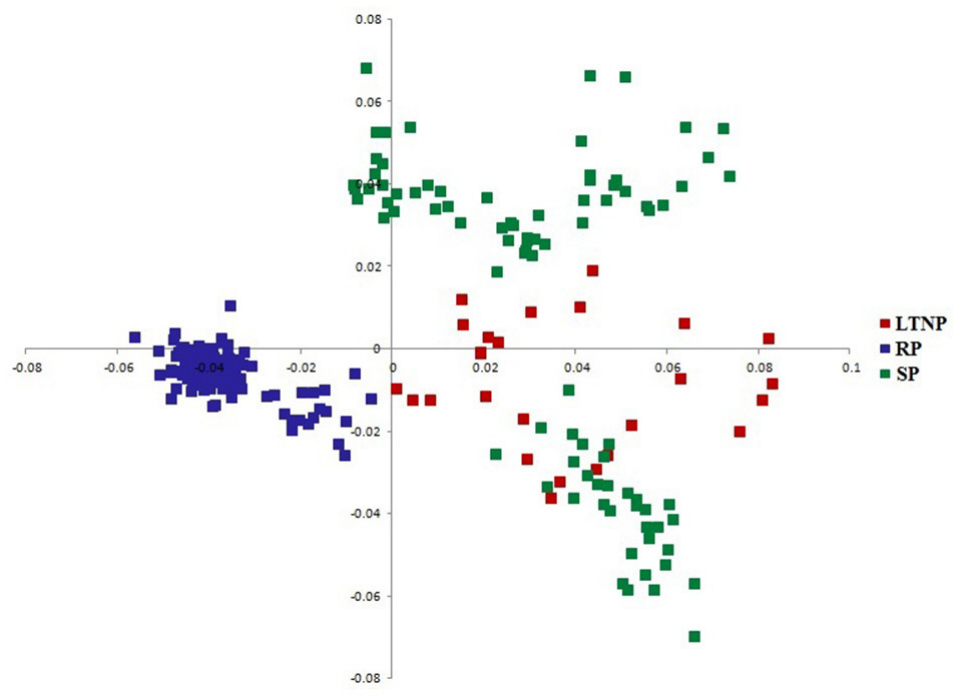
Distribution of env genes along the two major axes of correspondence analysis (COA) based on RAAU data. x-axis- Axis 1 of RAAU; y-axis- Axis 2 of RAAU. Red colored square boxes represent Long term non-progressor (LTNP) sequences. Blue colored square boxes depict Rapid progressor (RP) sequences. Green colored square boxes refer to Slow progressor (SP) sequences.

Correlating the observed phylogenetic profile of env genes with RAAU pattern appeared necessary in order to efficiently explore the impact of the various categories of env genes on gene sequence diversity. Accordingly, CoA on amino acid usage was performed using 264 env gene sequences representing three different categories of disease progression genes of HIV-1 subtype B. COA generated two separate clusters (Figure 2). Figure 2 shows the distribution of the genes along two major axes of amino acid usage variation.

It was perceptible from the amino acid usage based COA that SP and LTNP sequences shared similar patterns of amino acid usage different from RP sequences which formed a discrete cluster along Axis 1 (Figure 2). It was apparent from Figure 2 that the three categories of env genes i.e., RP, SP, and LTNP, had their own characteristic pattern of amino acid usage and the env genes of SP exhibited greater resemblance with env genes of LTNP in terms of amino acid usage pattern. It was also clear that the results of COA were in absolute accordance with phylogenetic profile (Figure 1).

### Impact of evolutionary selection pressure on three categories of env genes

Earlier, Canducci et al. (2009) observed that evolutionary rate of HIV-1 env gene varies between NP and LTNP patients. The evolutionary rate (ω) for the env sequences of RP, SP, and LTNP type was found to correlate significantly with Axis 1 (*R* = −0.31, *P* < 0.01) of RAAU data. It indicates that evolutionary selection pressure significantly influenced the amino acid usage pattern of the three different types of env gene sequences.

**Figure 3:**
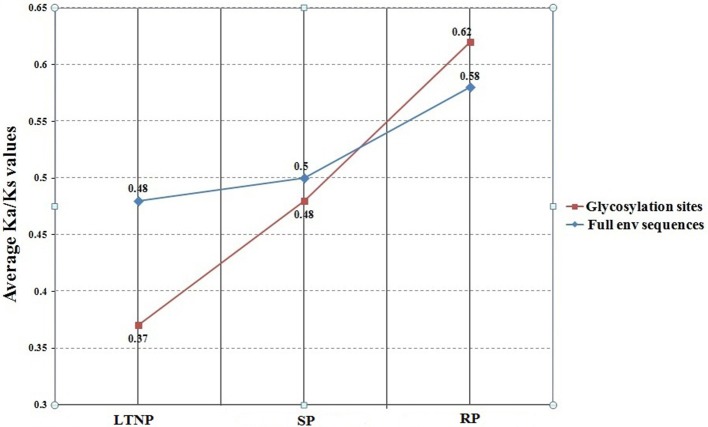
Variation of Ka/Ks of env genes (LTNP, SP and RP) and respective glycosylation sites. LTNP, Long term non-progressor; SP, Slow progressor; RP, Rapid progressor. The blue line refers to the plot of the average Ka/Ks values of the full env sequences of LTNP, SP, and RP. The red line represents the plot of the average Ka/Ks values of the associated glycosylation sites of LTNP, SP, and RP.

It was evident from COA of amino acid usage that env gene sequences belonging to the category SP and LTNP segregated from RP along Axis 1 of RAAU. Significant negative correlation of ω (Ka/Ks) with Axis 1 of RAAU indicated that the average Ka/Ks values of SP and LTNP sequences would be lesser than the average Ka/Ks value of RP sequences. It was indeed found that the average Ka/Ks value of LTNP sequences (0.48) was significantly less than that of average Ka/Ks value of RP sequences (0.58) (*P* < 0.01). We also observed that the average Ka/Ks value of SP sequences (0.49) was significantly less than that of RP sequences (0.58) (*P* < 0.01). But, the difference between the average Ka/Ks values of LTNP and SP sequences was observed to be statistically insignificant. Considering the fact that evolutionary rate might vary depending on the functional constraints, SWAKK web server (http://ibl.mdanderson.org/swakk/) was employed to calculate the evolutionary rate for each single amino acid residue in the concerned protein sequences. Extensive glycosylation of HIV-1 envelope proteins (env) is known to play an important role in evasion of host immune response. Average Ka/Ks value for the glycosylation sites of RP sequences were compared with that of the average Ka/Ks values of the LTNP and SP sequences. Interestingly, it was noted that the average Ka/Ks values for the glycosylation sites of LTNP (0.37) and SP (0.48) sequences were significantly less than that of the average Ka/Ks value of RP sequences (0.62) (*P* < 0.01). It was also noteworthy that the average Ka/Ks value of glycosylation sites for LTNP sequences was significantly less than that of SP sequences (*P* < 0.01; Figure 3). Such an observation clearly indicates the functional importance and vitality of glycosylation sites in case of HIV infection.

### Influence of host machinery on the disease progression of HIV based on RSCU

**Figure 4:**
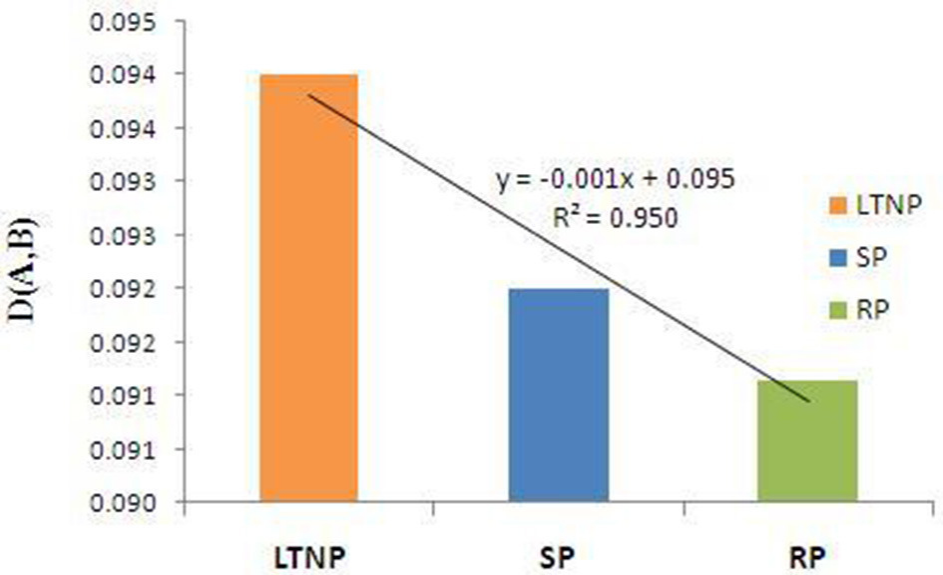
Similarity index [D(A,B)] of env genes (LTNP, SP, and RP) with respect to human host. Orange bar refers to similarity index value [D(A,B)] of Long term non-progressor (LTNP) sequences. Blue bar represents the similarity index value [D(A,B)] of the Slow progressor (SP) sets. Green bar depicts the similarity index value [D(A,B)] of the Rapid progressor (RP) sequences.

In the present study we have considered the similarity index parameter (Materials and Methods) to understand the influence of host genome on the adaptability of virus genome inside the host. Methodical inspection of similarity index of the three concerned categories of env gene sequences with human host revealed that the selection pressure due to human host was more severe on the LTNP sequences in comparison to the SP and RP sequence sets (Figure 4). Host associated selective constraints appeared most relaxed on the RP sequences as has been evident from Figure 4. Difference between similarity index values of the respective LTNP, SP, and RP sequences appeared to be statistically significant and the pattern of variation among them seemed distinct enough to infer that the LTNP sequences were under a stronger selective impact of human host.

Apart from estimating similarity index, we extended our analysis pertaining to the usage of identical codons among the three diverse types of env gene sequences with human host. Codons were defined as over-represented (RSCU > 1.6) and under-represented (RSCU < 0.6) as per scheme followed by Wong et al. (2010). Similar codon usage pattern was inferred when a particular codon was found to display RSCU values < 0.6 or more than 1.6 or found to fall within a range of 0.6 to 1.6 for both human host and the respective env type i.e., RP, SP, and LTNP. Interestingly, it was evident after careful inspection that LTNP sequences shared the highest frequency of similarly selected codons (36 codons out of 59 codons) with human host system in comparison to SP (34 codons out of 59 codons) and RP (32 codons out of 59 codons) types (Supplementary Table 1). Occurrence of higher frequencies of identically shared codons with human host in LTNP might eventually lead to a better adaptability and longer abode of the concerned LTNP in human cellular environment.

## Discussion

Intragenomic and intergenomic variations pertaining to codon and amino acid usage patterns can be well explained in the light of multivariate statistical analysis. Correspondence analysis (CoA) is one such type of comprehensive statistical tool which highlights the major variations among codon and amino acid usage data and places them in accordance with such observed variations (Greenacre, 1984). CoA on the basis of RAAU of the three concerned types of env gene sequences revealed their unique amino acid compositional features and separated them according to their diverse compositional traits along the two principle axes of separation of genes i.e., Axis 1 and Axis 2 of RAAU data (Figure 2). SP and LTNP sequences, to certain extent, had identical amino acid usage features as was reflected from COA on RAAU data (Figure 2). However, RP sequences clustered separately along Axis 1 signifying completely diverse amino acid usage patterns from SP and LTNP sequences. Such contrasting features of amino acid composition of RP sequences might have an implication in their rapid rate of disease progression in human host, characterized by a short stay in host cellular environment and speedy proliferation and infection (Kumar, 2013; Jarrin et al., 2015).

Earlier reports suggested that the env proteins of LTNP were subject to purifying selection for their overall mutability and tendencies of variation in LTNP. The impact of stronger purifying selection on env genes of LTNP in contrast to RP might be an outcome of the survival value conferred by adaptive stability of the envelop protein. To further validate our observation, we then fitted linear regression lines between Ka and Ks through the origin assuming that Ka and Ks should both initially be zero at the moment of lineage divergence. The slopes for the lines of whole env genes for LTNP and RP were found to be 0.48 and 0.58, respectively. These results indicated that the increase in Ka with respect to Ks in RP was around 1.2 times faster than in LTNP. Such observations were suggestive of the fact that env proteins of LTNP genes have been subject to more severe evolutionary selection with respect to RP, which eventually has been reflected at a lower level of non-synonymous nucleotide substitution for a given level of synonymous substitution. Similarly, the slope for SP was found to be 0.49, suggesting that the increase in Ka with respect to Ks in SP was around 1.01 times faster than in LTNP. This, in turn, reinforced the hypothesis that unusual functional constraints have been instrumental on the env protein sequences of LTNP. Higher average Ka/Ks value of RP over SP and LTNP sequences (statistically significant) indicated that the evolutionary constraint on the RP sequences has been lesser compared to the impact of evolutionary forces on the SP and LTNP sequences. Such an instance of relaxed selection pressure might confer an added advantage to the RP sequences, associated with rapid infection in a short span of time, in accumulation of non-synonymous mutations and subsequent evasion of host (human) immune response (Khanlou et al., 1996; Jarrin et al., 2015).

HIV-1 shows variation in susceptibility of infection to human (Fellay et al., 2007). Many attempts have been made to understand the viral genetics associated with disease progression in HIV-1 affected patients. However, molecular mechanism and host genetic influence on effective viral pathogenesis and subsequent progression of disease still remain unclear (Michael, 1999; Carrington et al., 2001).

Viruses mimic the codon usage profile of their respective hosts and rely largely on the chaperon apparatus of their hosts for proficient replication and enhanced robustness (Kunec and Osterrieder, 2016). In this pretext, similarity index of a concerned virus with its respective host has been suggested to be an effective tool to properly assess the impact of host genomic influence on the patterns of associated viral codonic signatures. Several earlier studies have used similarity index to demonstrate the influence of host genome over the viral genome (Nasrullah et al., 2015; Butt et al., 2016). Estimation of similarity index with human host among the concerned types of env sequences associated with varying degrees of disease progression i.e., RP, SP, and LTNP signified the fact that the impact of selection by human host was most intense in LTNP compared to SP and RP. Such observations seemed logical in light of the fact that LTNP sequences reside in the human system for long and are characterized by high CD4+ and CD8+ T-cell counts (Zeller et al., 1996; Kumar, 2013). Thus, higher similarity index with human host in LTNP and usage of a higher proportion of identically selected codons with human host tend to be a consequence of prolonged residence and adaptive efficacy of LTNP in human body. In contrast, the instance of lower similarity index in RP with human host might be well explained from the viewpoint that RP stays in human body for a shorter span and causes rapid infection (Khanlou et al., 1996). However, SP sequences displayed higher similarity index with human host in comparison to RP which might be attributed to a comparatively longer residence and slower disease progression than RP (Hogervorst et al., 1995). Thus, observations pertaining to similarity index among the three types of env sequences with human host seemed well correlated with the rates of disease progression and duration of abode in human host.

## Conclusion

Decreased HIV-1 strain evolution correlates with weaker viral fitness and the inability to evade the host immune system. Some studies have shown that viral strains from LTNPs are less evolved and thus, less capable of evading the host immunological response when compared with progressor strains (Wang et al., 2003; Sandonís et al., 2009). In our study, Ka/Ks decreased significantly for LTNP env gene sequences compared to RP gene sequences. Similar trend was observed for glycosylation sites of the respective sequences. Findings of the present study confirm that HIV-1 env gene of LTNP have been under stronger purifying selection compared to the respective RP counterpart. The env genes, coding for the env glycoproteins, experience severe selection constraints from the host due to their constant exposure to the host immune system. The env protein mediates HIV-1 entry into host CD4+ T cells. Reduction in Ka/Ks for env genes of LTNP might result in significantly slower infection of CD4+ T cells compared with RP. Our hypothesis seems well justified in light of the fact that the env protein plays essential role in receptor binding and cell entry (Wyatt and Sodroski, 1998) and efficiently maintains extensive glycosylation on its surface to avoid antibody neutralization (Reitter et al., 1998). In this perspective, it might be suggested that env gene evolution occurs mainly by negative selection with the occurrence of mutation that might not reach fixation in the viral population.

Present study addresses an evolutionary perspective that recognizes the ongoing dynamic interplay between human host and genetic diversity of HIV. Our analysis also provides an elucidation of the driving force accountable for the selection of favorable host genetic profiles as well as for viral mutations that are best adapted to the concerned host effects. Outcomes of the current research work confer a vivid portrait about the role of host factors in determining the overall progression of HIV infection.

## Author contributions

SB planned and designed the work. AR and RB generated the data. SB, AR, and RB analyzed data and prepared the manuscript. All authors read and approved the final manuscript.

### Conflict of interest statement

The authors declare that the research was conducted in the absence of any commercial or financial relationships that could be construed as a potential conflict of interest.
