# Supplementary material for: HIV Progression Depends on Codon and Amino Acid Usage Profile of Envelope Protein and Associated Host-Genetic Influence
Source: Front Microbiol. 2017 Jun 15;8:1083. doi: 10.3389/fmicb.2017.01083 (PMC5471322; doi:10.3389/fmicb.2017.01083)
Supplement: Supplementary file 1 [file Table1.DOC]

**Supplementary Table 1:** Relative Synonymous Codon Usage (RSCU) values of env genes (LTNP, SP and RP) with respect to human host.

|  |  | **HIV** | | | **Host** |  |  | **HIV** | | | **Host** |
| --- | --- | --- | --- | --- | --- | --- | --- | --- | --- | --- | --- |
| **AA** | **Codon** | **LTNP** | **SP** | **RP** | **HS** | **AA** | **Codon** | **LTNP** | **SP** | **RP** | **HS** |
| Phe | **UUU** | **1.10** | **1.19** | **1.08** | **0.93** | Ser | **UCU** | **0.88** | **0.75** | **0.89** | **1.13** |
|  | **UUC** | **0.90** | **0.81** | **0.92** | **1.07** |  | UCC | 0.32 | 0.41 | 0.39 | 1.31 |
| Leu | UUA | 1.41 | 1.45 | 1.35 | 0.46 |  | **UCA** | **1.24** | **1.14** | **1.28** | **0.90** |
|  | **UUG** | **1.30** | **1.26** | **1.26** | **0.77** |  | **UCG** | **0.22** | **0.23** | **0.27** | **0.33** |
|  | CUU | 0.55 | 0.48 | 0.60 | 0.79 |  | AGU | 2.00 | 2.16 | 2.04 | 0.90 |
|  | **CUC** | **0.93** | **0.96** | **0.99** | **1.17** |  | **AGC** | **1.35** | **1.32** | **1.13** | **1.44** |
|  | CUA | 0.83 | 0.84 | 0.72 | 0.43 | Arg | AGA | 3.92 | 3.81 | 3.80 | 1.29 |
|  | CUG | 0.98 | 1.02 | 1.05 | 2.37 |  | **CGU** | **0.01** | **0.05** | **0.01** | **0.48** |
| Ile | **AUU** | **0.97** | **1.04** | **0.95** | **1.08** |  | CGC | 0.29 | 0.36 | 0.31 | 1.10 |
|  | AUC | 0.48 | 0.43 | 0.51 | 1.41 |  | CGA | 0.05 | 0.12 | 0.11 | 0.65 |
|  | AUA | 1.55 | 1.53 | 1.54 | 0.51 |  | CGG | 0.14 | 0.15 | 0.2 | 1.21 |
| Val | GUU | 0.47 | 0.57 | 0.46 | 0.73 |  | **AGG** | **1.59** | **1.50** | **1.56** | **1.27** |
|  | GUC | 0.54 | 0.54 | 0.46 | 0.95 | Cys | **UGU** | **1.40** | **1.34** | **1.47** | **0.91** |
|  | GUA | 2.02 | 1.91 | 2.02 | 0.47 |  | **UGC^**^** | **0.61** | **0.66** | 0.53 | **1.09** |
|  | GUG | 0.97 | 0.98 | 1.06 | 1.85 | His | **CAU** | **1.22** | **1.33** | **1.41** | **0.84** |
| Pro | **CCU** | **0.85** | **0.78** | **0.87** | **1.15** |  | **CAC** | **0.78** | **0.67** | **0.59** | **1.16** |
|  | **CCC** | **1.04** | **1.15** | **1.05** | **1.29** | Gln | CAA | 1.03 | 1.04 | 0.99 | 0.53 |
|  | CCA | 1.88 | 1.86 | 1.68 | 1.11 |  | **CAG** | **0.97** | **0.96** | **1.01** | **1.47** |
|  | **CCG** | **0.23** | **0.20** | **0.40** | **0.45** | Asn | **AAU** | **1.45** | **1.48** | **1.44** | **0.94** |
| Thr | **ACU** | **1.13** | **1.22** | **1.01** | **0.99** |  | AAC | 0.55 | 0.52 | 0.56 | 1.06 |
|  | **ACC** | **0.95** | **0.87** | **1.03** | **1.42** | Lys | **AAA** | **1.30** | **1.26** | **1.32** | **0.87** |
|  | ACA | 1.65 | 1.62 | 1.62 | 1.14 |  | **AAG** | **0.70** | **0.74** | **0.68** | **1.13** |
|  | **ACG** | **0.28** | **0.28** | **0.33** | **0.46** | Asp | **GAU** | **1.17** | **1.22** | **1.24** | **0.93** |
| Ala | **GCU** | **1.18** | **1.14** | **1.17** | **1.06** |  | **GAC** | **0.83** | **0.78** | **0.76** | **1.07** |
|  | **GCC** | **0.84** | **0.70** | **0.82** | **1.60** | Glu | **GAA** | **1.49** | **1.52** | **1.53** | **0.84** |
|  | **GCA^*^** | **1.60** | 1.77 | 1.75 | **0.91** |  | GAG | 0.51 | 0.48 | 0.47 | 1.16 |
|  | **GCG** | **0.31** | **0.38** | **0.26** | **0.42** | Gly | **GGU^**^** | **0.61** | **0.68** | 0.57 | **0.65** |
| Tyr | **UAU** | **1.41** | **1.43** | **1.43** | **0.89** |  | GGC | 0.48 | 0.49 | 0.44 | 1.35 |
|  | **UAC^*^** | **0.61** | 0.57 | 0.57 | **1.11** |  | GGA | 1.97 | 1.94 | 2.05 | 1.00 |
|  |  |  |  |  |  |  | **GGG** | **0.96** | **0.89** | **0.94** | **1.00** |

AA: Amino acid; LTNP: Long term non-progressor; SP: Slow progressor; RP: Rapid progressor; HS: *Homo sapiens*. Similarly selected codons between the three types of env gene sequences and human host are marked in bold. Identically selected codons, exclusively between LTNP sequences and human host, have been marked ^*^. Similarly employed codons between LTNP and SP sequences, but not RP sets, with human host have been marked ^**^.
