# Supplementary material for: HIV Progression Depends on Codon and Amino Acid Usage Profile of Envelope Protein and Associated Host-Genetic Influence
Source: Front Microbiol. 2017 Jun 15;8:1083. doi: 10.3389/fmicb.2017.01083 (PMC5471322; doi:10.3389/fmicb.2017.01083)
Supplement: Supplementary file 2 [file DataSheet1.DOC]

B.GB.1997.CW010.AJ418486LTNP_SUBTYPE_B

B.GB.1997.CW010.AJ418487LTNP_SUBTYPE_B

B.GB.1997.CW010.AJ418488LTNP_SUBTYPE_B

B.ES.-.LMP0503.DQ141343LTNP_SUBTYPE_B

B.US.2003.WC3_0603.EF121408LTNP_SUBTYPE_B

B.ES.-.MH01.EF531330LTNP_SUBTYPE_B

B.ES.-.MH02.EF531331LTNP_SUBTYPE_B

B.US.2006.2857_p1.FJ152546LTNP_SUBTYPE_B

B.US.2006.2866_P4.FJ152547LTNP_SUBTYPE_B

B.NL.1985.19642_3_1G11.GU455425LTNP_SUBTYPE_B

B.NL.1985.19642_3_2F1.GU455427LTNP_SUBTYPE_B

B.NL.1985.19642_3_2G8.GU455428LTNP_SUBTYPE_B

B.NL.1987.19642_10_1E4.GU455432LTNP_SUBTYPE_B

B.NL.1987.19642_10_1F3.GU455433LTNP_SUBTYPE_B

B.NL.1987.19642_10_2C6.GU455435LTNP_SUBTYPE_B

B.NL.1989.19642_18_1C7.GU455437LTNP_SUBTYPE_B

B.NL.1989.19642_18_1G7.GU455440LTNP_SUBTYPE_B

B.NL.1992.19642_29_1C8.GU455442LTNP_SUBTYPE_B

B.NL.1992.19642_29_1D11.GU455443LTNP_SUBTYPE_B

B.NL.1992.19642_29_1F5.GU455444LTNP_SUBTYPE_B

B.NL.1992.19642_29_1G4.GU455445LTNP_SUBTYPE_B

B.NL.1992.19642_29_2F6.GU455446LTNP_SUBTYPE_B

B.NL.1994.19642_38_2A6.GU455447LTNP_SUBTYPE_B

B.NL.1994.19642_38_1G7.GU455448LTNP_SUBTYPE_B

B.NL.1995.19642_41_1A3.GU455451LTNP_SUBTYPE_B

B.NL.1995.19642_41_1F6.GU455452LTNP_SUBTYPE_B

B.NL.1995.19642_41_1H1.GU455453LTNP_SUBTYPE_B

B.ES.-.B_ES_MH01.KC595179LTNP_SUBTYPE_B

B.ES.-.B_ES_MH02.KC595180LTNP_SUBTYPE_B

B.ES.2004.RF_LTNP_03.KC595193LTNP_SUBTYPE_B

B.GB.1996.875DEN49.AJ535612Rapid_Progressor_SUBTYPE_B

B.GB.1996.875DEN51.AJ535613Rapid_Progressor_SUBTYPE_B

B.US.1985.CC1_85_7.AY357341Rapid_Progressor_SUBTYPE_B

B.US.1985.CC1_85_9.AY357342Rapid_Progressor_SUBTYPE_B

B.US.1985.CC1_85_10.AY357343Rapid_Progressor_SUBTYPE_B

B.US.1985.CC1_85_8.AY357344Rapid_Progressor_SUBTYPE_B

B.US.1985.CC1_85_11.AY357345Rapid_Progressor_SUBTYPE_B

B.US.1985.CC101_2_12.AY357350Rapid_Progressor_SUBTYPE_B

B.US.1985.CC101_2_4.AY357352Rapid_Progressor_SUBTYPE_B

B.US.1985.CC101_2_11.AY357353Rapid_Progressor_SUBTYPE_B

B.US.1985.CC101_2_13.AY357354Rapid_Progressor_SUBTYPE_B

B.US.1985.CC101_2_5.AY357355Rapid_Progressor_SUBTYPE_B

B.US.1985.CC101_4_14.AY357356Rapid_Progressor_SUBTYPE_B

B.US.1985.CC101_4_13.AY357357Rapid_Progressor_SUBTYPE_B

B.US.1985.CC101_4_9.AY357358Rapid_Progressor_SUBTYPE_B

B.US.1985.CC101_4_12.AY357359Rapid_Progressor_SUBTYPE_B

B.US.1985.CC101_4_1.AY357360Rapid_Progressor_SUBTYPE_B

B.US.1985.CC101_4_5.AY357361Rapid_Progressor_SUBTYPE_B

B.US.1985.CC101_4_10.AY357362Rapid_Progressor_SUBTYPE_B

B.US.1985.CC101_4_6.AY357365Rapid_Progressor_SUBTYPE_B

B.US.1985.CC101_4_8.AY357366Rapid_Progressor_SUBTYPE_B

B.US.1985.CC101_4_7.AY357367Rapid_Progressor_SUBTYPE_B

B.US.1985.CC101_6_1.AY357368Rapid_Progressor_SUBTYPE_B

B.US.1985.CC101_6_10.AY357369Rapid_Progressor_SUBTYPE_B

B.US.1985.CC101_6_2.AY357371Rapid_Progressor_SUBTYPE_B

B.US.1985.CC101_6_4.AY357373Rapid_Progressor_SUBTYPE_B

B.US.1985.CC101_6_5.AY357374Rapid_Progressor_SUBTYPE_B

B.US.1985.CC101_6_7.AY357376Rapid_Progressor_SUBTYPE_B

B.US.1985.CC101_6_8.AY357377Rapid_Progressor_SUBTYPE_B

B.US.1985.CC101_6_9.AY357378Rapid_Progressor_SUBTYPE_B

B.US.1985.CC101_10_1.AY357398Rapid_Progressor_SUBTYPE_B

B.US.1985.CC101_10_2.AY357399Rapid_Progressor_SUBTYPE_B

B.US.1985.CC101_10_4.AY357401Rapid_Progressor_SUBTYPE_B

B.US.1985.CC101_10_7.AY357403Rapid_Progressor_SUBTYPE_B

B.US.1985.CC101_10_8.AY357404Rapid_Progressor_SUBTYPE_B

B.US.1985.CC101_10_9.AY357405Rapid_Progressor_SUBTYPE_B

B.US.1985.CC101_10_11.AY357407Rapid_Progressor_SUBTYPE_B

B.US.1985.CC101_10_12.AY357408Rapid_Progressor_SUBTYPE_B

B.US.1985.CC101_10_13.AY357409Rapid_Progressor_SUBTYPE_B

B.US.1985.CC101_10_14.AY357410Rapid_Progressor_SUBTYPE_B

B.US.1985.CC101_12_9.AY357411Rapid_Progressor_SUBTYPE_B

B.US.1985.CC101_12_7.AY357413Rapid_Progressor_SUBTYPE_B

B.US.1985.CC101_12_4.AY357414Rapid_Progressor_SUBTYPE_B

B.US.1985.CC101_12_8.AY357415Rapid_Progressor_SUBTYPE_B

B.US.1985.CC101_12_13.AY357416Rapid_Progressor_SUBTYPE_B

B.US.1985.CC101_12_12.AY357418Rapid_Progressor_SUBTYPE_B

B.US.1985.CC101_12_6.AY357419Rapid_Progressor_SUBTYPE_B

B.US.1985.CC101_12_5.AY357420Rapid_Progressor_SUBTYPE_B

B.US.1985.CC101_12_3.AY357422Rapid_Progressor_SUBTYPE_B

B.US.1985.CC101_12_2.AY357423Rapid_Progressor_SUBTYPE_B

B.US.1985.CC101_14_3.AY357424Rapid_Progressor_SUBTYPE_B

B.US.1985.CC101_14_14.AY357425Rapid_Progressor_SUBTYPE_B

B.US.1985.CC101_14_1.AY357427Rapid_Progressor_SUBTYPE_B

B.US.1985.CC101_14_18.AY357428Rapid_Progressor_SUBTYPE_B

B.US.1985.CC101_14_17.AY357429Rapid_Progressor_SUBTYPE_B

B.US.1985.CC101_14_16.AY357431Rapid_Progressor_SUBTYPE_B

B.US.1985.CC101_14_15.AY357435Rapid_Progressor_SUBTYPE_B

B.US.1985.CC101_14_5.AY357436Rapid_Progressor_SUBTYPE_B

B.US.1985.CC101_14_13.AY357438Rapid_Progressor_SUBTYPE_B

B.US.1985.CC101_14_19.AY357439Rapid_Progressor_SUBTYPE_B

B.US.1985.CC101_14_7.AY357440Rapid_Progressor_SUBTYPE_B

B.US.1985.CC101_16_6.AY357441Rapid_Progressor_SUBTYPE_B

B.US.1985.CC101_16_8.AY357444Rapid_Progressor_SUBTYPE_B

B.US.1985.CC101_16_7.AY357445Rapid_Progressor_SUBTYPE_B

B.US.1985.CC101_16_2.AY357446Rapid_Progressor_SUBTYPE_B

B.US.1985.CC101_16_12.AY357447Rapid_Progressor_SUBTYPE_B

B.US.1985.CC101_16_4.AY357448Rapid_Progressor_SUBTYPE_B

B.US.1985.CC101_16_10.AY357450Rapid_Progressor_SUBTYPE_B

B.US.1985.CC101_16_11.AY357451Rapid_Progressor_SUBTYPE_B

B.US.1985.CC101_16_5.AY357452Rapid_Progressor_SUBTYPE_B

B.US.1985.CC101_18_8.AY357453Rapid_Progressor_SUBTYPE_B

B.US.1985.CC101_18_6.AY357454Rapid_Progressor_SUBTYPE_B

B.US.1985.CC101_18_1.AY357455Rapid_Progressor_SUBTYPE_B

B.US.1985.CC101_18_7.AY357456Rapid_Progressor_SUBTYPE_B

B.US.1985.CC101_18_11.AY357457Rapid_Progressor_SUBTYPE_B

B.US.1985.CC101_18_4.AY357458Rapid_Progressor_SUBTYPE_B

B.US.1985.CC101_18_3.AY357459Rapid_Progressor_SUBTYPE_B

B.US.1985.CC101_18_10.AY357460Rapid_Progressor_SUBTYPE_B

B.US.1985.CC101_18_5.AY357461Rapid_Progressor_SUBTYPE_B

B.US.1985.CC101_18_2.AY357462Rapid_Progressor_SUBTYPE_B

B.US.1985.CC101_18_9.AY357463Rapid_Progressor_SUBTYPE_B

B.US.1985.CC101_19_18.AY357464Rapid_Progressor_SUBTYPE_B

B.US.1985.CC101_19_7.AY357465Rapid_Progressor_SUBTYPE_B

B.US.1985.CC101_19_3.AY357466Rapid_Progressor_SUBTYPE_B

B.US.1985.CC101_19_4.AY357467Rapid_Progressor_SUBTYPE_B

B.US.1985.CC101_19_15.AY357468Rapid_Progressor_SUBTYPE_B

B.US.1985.CC101_19_20.AY357469Rapid_Progressor_SUBTYPE_B

B.US.1985.CC101_20_2.AY357471Rapid_Progressor_SUBTYPE_B

B.US.1985.CC101_20_1.AY357474Rapid_Progressor_SUBTYPE_B

B.US.1985.CC101_20_7.AY357475Rapid_Progressor_SUBTYPE_B

B.US.1985.CC101_20_11.AY357476Rapid_Progressor_SUBTYPE_B

B.US.1985.CC101_20_6.AY357477Rapid_Progressor_SUBTYPE_B

B.US.1985.CC101_20_3.AY357478Rapid_Progressor_SUBTYPE_B

B.US.1985.CC101_20_10.AY357479Rapid_Progressor_SUBTYPE_B

B.US.1985.CC101_20_5.AY357480Rapid_Progressor_SUBTYPE_B

B.US.1985.CC101_22R2_3.AY357481Rapid_Progressor_SUBTYPE_B

B.US.1985.CC101_22R2_6.AY357482Rapid_Progressor_SUBTYPE_B

B.US.1985.CC101_22R2_9.AY357483Rapid_Progressor_SUBTYPE_B

B.US.1985.CC101_22R2_4.AY357484Rapid_Progressor_SUBTYPE_B

B.US.1985.CC101_22R2_12.AY357485Rapid_Progressor_SUBTYPE_B

B.US.1985.CC101_22R2_11.AY357486Rapid_Progressor_SUBTYPE_B

B.US.1985.CC101_22R2_8.AY357487Rapid_Progressor_SUBTYPE_B

B.US.1985.CC101_22R2_5.AY357488Rapid_Progressor_SUBTYPE_B

B.US.1985.CC101_22R2_2.AY357489Rapid_Progressor_SUBTYPE_B

B.US.1985.CC101_22R2_7.AY357490Rapid_Progressor_SUBTYPE_B

B.US.1985.CC101_22R2_1.AY357491Rapid_Progressor_SUBTYPE_B

B.US.1985.CC101_22R5_11.AY357494Rapid_Progressor_SUBTYPE_B

B.US.1985.CC101_22R5_10.AY357495Rapid_Progressor_SUBTYPE_B

B.US.1985.CC101_22R5_8.AY357497Rapid_Progressor_SUBTYPE_B

B.US.1985.CC101_22R5_5.AY357498Rapid_Progressor_SUBTYPE_B

B.US.1985.CC101_22R5_2.AY357500Rapid_Progressor_SUBTYPE_B

B.US.1985.CC101_22R5_9.AY357501Rapid_Progressor_SUBTYPE_B

B.US.1985.CC101_22R5_3.AY357503Rapid_Progressor_SUBTYPE_B

B.US.1985.CC101_22R9_11.AY357504Rapid_Progressor_SUBTYPE_B

B.US.1985.CC101_22R9_2.AY357505Rapid_Progressor_SUBTYPE_B

B.US.1985.CC101_22R9_5.AY357506Rapid_Progressor_SUBTYPE_B

B.US.1985.CC101_22R9_10.AY357507Rapid_Progressor_SUBTYPE_B

B.US.1986.CC2_86_9.AY357550Rapid_Progressor_SUBTYPE_B

B.US.1986.CC2_86_10.AY357551Rapid_Progressor_SUBTYPE_B

B.US.1986.CC2_86_11.AY357552Rapid_Progressor_SUBTYPE_B

B.US.1986.CC2_86_2.AY357553Rapid_Progressor_SUBTYPE_B

B.US.1986.CC2_86_6.AY357554Rapid_Progressor_SUBTYPE_B

B.US.1986.CC2_86_12.AY357555Rapid_Progressor_SUBTYPE_B

B.US.1986.CC7_86_5.AY357557Rapid_Progressor_SUBTYPE_B

B.US.1986.CC7_86_11.AY357558Rapid_Progressor_SUBTYPE_B

B.US.1986.CC7_86_9.AY357559Rapid_Progressor_SUBTYPE_B

B.US.1986.CC7_86_4.AY357560Rapid_Progressor_SUBTYPE_B

B.US.1986.CC7_86_1.AY357562Rapid_Progressor_SUBTYPE_B

B.US.1986.CC7_86_2.AY357564Rapid_Progressor_SUBTYPE_B

B.US.1986.CC7_86_3.AY357565Rapid_Progressor_SUBTYPE_B

B.US.1986.CC7_86_12.AY357566Rapid_Progressor_SUBTYPE_B

B.US.2001.45_01dTB13.JQ609684Slow_Progressor_SUBTYPE_B

B.US.2001.45_01dG18.JQ609696Slow_Progressor_SUBTYPE_B

B.US.2001.45_01dG21.JQ609699Slow_Progressor_SUBTYPE_B

B.US.2001.45_01dH9.JQ609707Slow_Progressor_SUBTYPE_B

B.US.2001.45_01B20.JQ609734Slow_Progressor_SUBTYPE_B

B.US.2001.45_01B25.JQ609738Slow_Progressor_SUBTYPE_B

B.US.2001.45_01D2.JQ609742Slow_Progressor_SUBTYPE_B

B.US.2006.45_06A2.JQ609784Slow_Progressor_SUBTYPE_B

B.US.2006.45_06A3.JQ609785Slow_Progressor_SUBTYPE_B

B.US.2006.45_06A4.JQ609786Slow_Progressor_SUBTYPE_B

B.US.2006.45_06A6.JQ609787Slow_Progressor_SUBTYPE_B

B.US.2006.45_06A7.JQ609788Slow_Progressor_SUBTYPE_B

B.US.2006.45_06A8.JQ609789Slow_Progressor_SUBTYPE_B

B.US.2006.45_06A10.JQ609791Slow_Progressor_SUBTYPE_B

B.US.2006.45_06B5.JQ609794Slow_Progressor_SUBTYPE_B

B.US.2006.45_06B14.JQ609798Slow_Progressor_SUBTYPE_B

B.US.2006.45_06C7.JQ609812Slow_Progressor_SUBTYPE_B

B.US.2006.45_06C8.JQ609813Slow_Progressor_SUBTYPE_B

B.US.2009.45_09A7.JQ609822Slow_Progressor_SUBTYPE_B

B.US.2009.45_09A9.JQ609824Slow_Progressor_SUBTYPE_B

B.US.2009.45_09A17.JQ609830Slow_Progressor_SUBTYPE_B

B.US.1995.1_95TC14.JQ609870Slow_Progressor_SUBTYPE_B

B.US.1995.1_95TC1.JQ609871Slow_Progressor_SUBTYPE_B

B.US.1995.1_95A10.JQ609872Slow_Progressor_SUBTYPE_B

B.US.1995.1_95A20.JQ609873Slow_Progressor_SUBTYPE_B

B.US.1995.1_95TC4.JQ609875Slow_Progressor_SUBTYPE_B

B.US.1995.1_95C18.JQ609876Slow_Progressor_SUBTYPE_B

B.US.1995.1_95A14.JQ609877Slow_Progressor_SUBTYPE_B

B.US.1995.1_95C10.JQ609878Slow_Progressor_SUBTYPE_B

B.US.1995.1_95C17.JQ609879Slow_Progressor_SUBTYPE_B

B.US.1995.1_95TC8.JQ609880Slow_Progressor_SUBTYPE_B

B.US.1995.1_95TC9.JQ609881Slow_Progressor_SUBTYPE_B

B.US.1995.1_95TC11.JQ609882Slow_Progressor_SUBTYPE_B

B.US.1995.1_95TC12.JQ609883Slow_Progressor_SUBTYPE_B

B.US.1995.1_95A15.JQ609884Slow_Progressor_SUBTYPE_B

B.US.1995.1_95A17.JQ609885Slow_Progressor_SUBTYPE_B

B.US.1995.1_95A18.JQ609886Slow_Progressor_SUBTYPE_B

B.US.1995.1_95A19.JQ609887Slow_Progressor_SUBTYPE_B

B.US.1995.1_95C2.JQ609888Slow_Progressor_SUBTYPE_B

B.US.1995.1_95C6.JQ609889Slow_Progressor_SUBTYPE_B

B.US.1995.1_95C12.JQ609890Slow_Progressor_SUBTYPE_B

B.US.1999.18_99A3.JQ609974Slow_Progressor_SUBTYPE_B

B.US.1999.18_99A5.JQ609975Slow_Progressor_SUBTYPE_B

B.US.1999.18_99A19.JQ609983Slow_Progressor_SUBTYPE_B

B.US.1999.18_99A25.JQ609987Slow_Progressor_SUBTYPE_B

B.US.1999.18_99A28.JQ609990Slow_Progressor_SUBTYPE_B

B.US.1999.18_99A29.JQ609991Slow_Progressor_SUBTYPE_B

B.US.1999.18_99A34.JQ609994Slow_Progressor_SUBTYPE_B

B.US.1999.18_99A37.JQ609996Slow_Progressor_SUBTYPE_B

B.US.1999.18_99A38.JQ609997Slow_Progressor_SUBTYPE_B

B.US.1999.18_99A41.JQ609999Slow_Progressor_SUBTYPE_B

B.US.1999.18_99A42.JQ610000Slow_Progressor_SUBTYPE_B

B.US.1999.18_99B1.JQ610001Slow_Progressor_SUBTYPE_B

B.US.1999.18_99B2.JQ610002Slow_Progressor_SUBTYPE_B

B.US.1999.18_99B4.JQ610003Slow_Progressor_SUBTYPE_B

B.US.1999.18_99B5.JQ610004Slow_Progressor_SUBTYPE_B

B.US.1999.18_99B7.JQ610005Slow_Progressor_SUBTYPE_B

B.US.1999.18_99B11.JQ610008Slow_Progressor_SUBTYPE_B

B.US.1999.18_99B15.JQ610011Slow_Progressor_SUBTYPE_B

B.US.1999.18_99B21.JQ610016Slow_Progressor_SUBTYPE_B

B.US.1999.18_99B24.JQ610018Slow_Progressor_SUBTYPE_B

B.US.2007.N26_07A1.JQ610053Slow_Progressor_SUBTYPE_B

B.US.2007.N26_07A2.JQ610054Slow_Progressor_SUBTYPE_B

B.US.2007.N26_07A4.JQ610056Slow_Progressor_SUBTYPE_B

B.US.2007.N26_07A6.JQ610057Slow_Progressor_SUBTYPE_B

B.US.2007.N26_07A7.JQ610058Slow_Progressor_SUBTYPE_B

B.US.2007.N26_07A8.JQ610059Slow_Progressor_SUBTYPE_B

B.US.2007.N26_07A10.JQ610060Slow_Progressor_SUBTYPE_B

B.US.2007.N26_07A16.JQ610062Slow_Progressor_SUBTYPE_B

B.US.2007.N26_07A19.JQ610063Slow_Progressor_SUBTYPE_B

B.US.2007.N26_07A21.JQ610064Slow_Progressor_SUBTYPE_B

B.US.2007.N26_07A22.JQ610065Slow_Progressor_SUBTYPE_B

B.US.2007.N26_07A31.JQ610068Slow_Progressor_SUBTYPE_B

B.US.2007.N26_07A34.JQ610069Slow_Progressor_SUBTYPE_B

B.US.2007.N26_07A38.JQ610070Slow_Progressor_SUBTYPE_B

B.US.2007.N26_07A39.JQ610071Slow_Progressor_SUBTYPE_B

B.US.2007.N26_07A40.JQ610072Slow_Progressor_SUBTYPE_B

B.US.2007.N26_07A41.JQ610073Slow_Progressor_SUBTYPE_B

B.US.2007.N26_07A42.JQ610074Slow_Progressor_SUBTYPE_B

B.US.2007.N26_07A45.JQ610075Slow_Progressor_SUBTYPE_B

B.US.2007.N26_07A48.JQ610076Slow_Progressor_SUBTYPE_B

B.US.2007.N26_07A50.JQ610077Slow_Progressor_SUBTYPE_B

B.US.2007.N26_07A51.JQ610078Slow_Progressor_SUBTYPE_B

B.US.2007.N26_07A53.JQ610079Slow_Progressor_SUBTYPE_B

B.US.2007.N26_07A55.JQ610080Slow_Progressor_SUBTYPE_B

B.US.2007.N26_07A57.JQ610081Slow_Progressor_SUBTYPE_B

B.US.2007.N26_07B1.JQ610082Slow_Progressor_SUBTYPE_B

B.US.2007.N26_07B5.JQ610083Slow_Progressor_SUBTYPE_B

B.US.2007.N26_07B6.JQ610084Slow_Progressor_SUBTYPE_B

B.US.2007.N26_07B9.JQ610085Slow_Progressor_SUBTYPE_B

B.US.2007.N26_07B10.JQ610086Slow_Progressor_SUBTYPE_B

B.US.2007.N26_07B12.JQ610087Slow_Progressor_SUBTYPE_B

B.US.2008.N90_08A6.JQ610123Slow_Progressor_SUBTYPE_B

B.US.2008.N90_08A9.JQ610125Slow_Progressor_SUBTYPE_B

B.US.2008.N90_08A20.JQ610133Slow_Progressor_SUBTYPE_B

B.US.2008.N90_08A28.JQ610137Slow_Progressor_SUBTYPE_B

B.US.2008.N90_08B2.JQ610139Slow_Progressor_SUBTYPE_B

B.US.2011.N152_061511_1.KM516886Slow_Progressor_SUBTYPE_B

B.US.2011.N152_061511_8.KM516887Slow_Progressor_SUBTYPE_B

B.US.2011.N152_061511_15.KM516888Slow_Progressor_SUBTYPE_B

B.US.2011.N152_061511_2.KM516889Slow_Progressor_SUBTYPE_B

B.US.2011.N152_061511_4.KM516890Slow_Progressor_SUBTYPE_B

B.US.2011.N152_061511_17.KM516891Slow_Progressor_SUBTYPE_B
